# 1190. Nudging Away from Alert Fatigue: A Novel Method for Tracking the Impact of Integrated Clinical Decision Support on Meropenem Order Rates

**DOI:** 10.1093/ofid/ofad500.1030

**Published:** 2023-11-27

**Authors:** Camille Ezran, Nicholas J Mercuro, Kristina E Rokas, Vince Dryer, Stephen Rawlings, Elizabeth Herrle

**Affiliations:** Maine Medical Center, Portland, Maine; Maine Medical Center, Portland, Maine; Maine Medical Center, Portland, Maine; Maine Medical Center, Portland, Maine; Maine Medical Center, Portland, Maine; Maine Medical Center, Portland, Maine

## Abstract

**Background:**

The electronic health record (EHR) has streamlined quality of care and hospital prescribing; however, interruptive alerts and hard restrictions on antibiotic ordering can disrupt clinical workflow, often without significant benefit. This study aimed to determine the impact of permissive restriction on meropenem orders with embedded clinical decision support and prescriber attestation.

**Methods:**

A quality improvement intervention implemented at Maine Medical Center (MMC, a 700-bed academic medical center) was evaluated through a multisite, quasiexperimental study within MaineHealth Hospitals. The intervention consisted of a provider-facing summary of guideline-directed indications for empiric and culture-directed uses of meropenem attached alongside the EHR order, and a required attestation button. To track meropenem order rates for adult patients prior to (Oct – Dec 2022) and after (Jan – Mar 2023) the intervention, the EHR was configured to fire a “silent” Best Practice Advisory behind-the-scenes when a provider opened the meropenem order. Rates of ordering were also monitored at other sites in MaineHealth, where the intervention was not implemented. The primary endpoint was the rate of “back-out” events, defined as a prescriber beginning a meropenem order but not signing it, and the secondary endpoint was the meropenem administration rate within 24 hours of opening an order.

**Results:**

A meropenem order was initiated in 824 unique encounters during the study period; following the intervention at MMC, the back-out rate for orders increased from 20.3% (n =291) to 32.7% (n= 147, p = .01), while there was no significant difference during the same period at institutions where the intervention was not implemented (Table 1). The rate of patients who received meropenem within 24 hours following an order being opened decreased from 90.0% pre-intervention to 74.8% post-intervention (p < 0.01).

**Figure:**
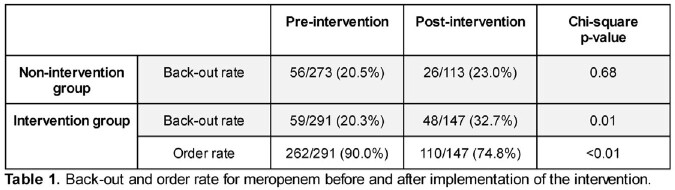

**Conclusion:**

This study suggests that simple, non-resource intensive antimicrobial stewardship ‘nudges’ can simultaneously be implemented and monitored through creative EHR design, leading to a reduction in overall completion of meropenem orders and a decrease in inappropriate antibiotic prescribing. Similar interventions can be implemented in all fields of healthcare stewardship.

**Disclosures:**

**All Authors**: No reported disclosures

